# Male song of the Aquatic Warbler, a promiscuous bird without paternal care, is more complex than previously thought

**DOI:** 10.1038/s41598-023-33001-9

**Published:** 2023-04-07

**Authors:** Tomasz S. Osiejuk, Justyna Kubacka

**Affiliations:** 1 Department of Behavioural Ecology, Institute of Environmental Biology, Faculty of Biology, Adam Mickiewicz University, Uniwersytetu Poznańskiego 6, 61-614 Poznań, Poland; 2 Museum and Institute of Zoology, Polish Academy of Sciences, Wilcza 64, 00-679 Warszawa, Poland

**Keywords:** Animal behaviour, Behavioural ecology

## Abstract

The Aquatic Warbler *Acrocephalus paludicola* is one of the rarest European passerines and is characterised by promiscuity, lack of pair-bonds and female-only parental care. This makes the species an important model for studying the function of avian courtship song. The song of the Aquatic Warbler consists of whistle and rattle phrases produced as discontinuous A-, B- and C-songs, which are built by a single rattle, a rattle and a whistle, and more than two phrases of both kinds, respectively. The A- and B-songs are thought to be aggressive signals in male-male interactions, while C-songs are thought to be important for female choice. Here, we analysed recordings of 40 individually marked males, and determined the phrase repertoire. The enumerated repertoire (males recorded for ≥ 10 min) ranged from 16 to 158 (mean 99), however, it did not capture the complete phrase repertoires. We then used models from species diversity ecology to estimate the actual phrase repertoire size, which ranged between 18 and 300 phrases (mean 155). The estimated repertoire was predicted by the number of C-songs. The rattle repertoire was larger than the whistle repertoire, and both positively correlated with the number of C-songs. Our study indicates that male Aquatic Warblers have highly complex phrase repertoires that vary widely in size. Their courtship song is flexible and efficient, enabling relative song complexity to be demonstrated in a short sample, thus facilitating both female attraction through the quick presentation of large phrase repertoires and rival deterrence through the production of many short and simple A- and B-songs.

## Introduction

### Bird song as a signal

Bird song is a signal that has evolved into an extremely rich set of structurally and functionally diversified forms. In temperate species, song is primarily a male trait and has two well recognised functions, territorial defence and mate attraction, which are often fulfilled together, albeit with varying degrees of emphasis^1^. These roles are typically performed by a repertoire of different vocal units (syllables or song types). The evolution of repertoires is therefore thought to be driven by intra- and inter-sexual selection. Repertoire size might serve as an honest signal of male quality and in several songbirds has been demonstrated to be an efficient female attractant. In polygynous species, larger repertoires can enable faster mating or the attraction of more females for copulation^2^.

Male bird song is often divided into two wide classes, continuous and discontinuous song. In continuous song, e.g. in the Skylark *Alauda arvensis*, males produce long sequences of syllables with short time gaps between them^3^. The discontinuous song, e.g. in the Yellowhammer *Emberiza citrinella*, song is organised in strophes that typically last a few seconds each, and the gaps between consecutive strophes are much longer than strophe duration^4^. Discontinuous singers usually have smaller repertoires than continuous ones, even if we consider syllables instead of strophes.

### Song of *Acrocephalus* warblers as a model

**Figure 1:**
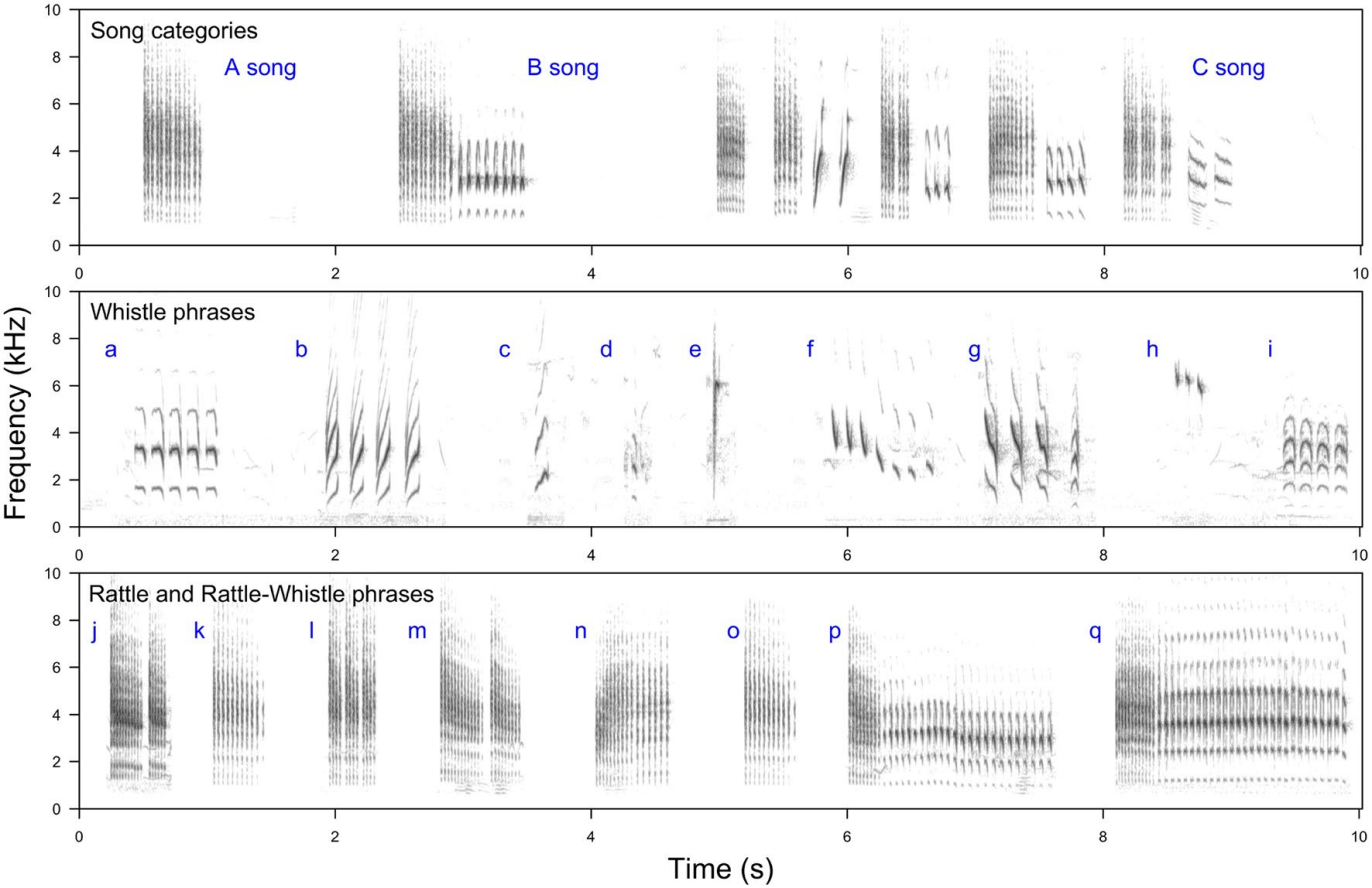
Sonograms illustrating variation of song of the Aquatic Warbler males. The ‘Song categories’ panel presents three basic categories of songs distinguished based on their structure: A-songs built of a single rattle phrase, B-songs built of rattles and whistles, and C-songs built of more than two rattles and whistles produced in a sequence. The ‘Whistle phrases’ panel presents the different whistles (**a**–**i**) which are characterised in the main text. Similarly, the ‘Rattle and Rattle–Whistle phrases’ panel presents variation of rattles (**j**–**o**) and mixed rattle-whistle phrases (**p**, **q**).

Continuous song has been studied using warblers of the genus *Acrocephalus* as models^1^. However, not all *Acrocephalus* warblers sing continuously, and some species are much more flexible in the organisation of song output. An example is the Aquatic Warbler *Acrocephalus paludicola*, one of the rarest European passerines. Due to limited and inaccessible breeding sites, its song is much less known compared to other acrocephalids. It has some common features (e.g. types of sounds produced) with other well-studied *Acrocephalus* warblers, but it also has some striking differences^5^. Males produce relatively short, repetitive songs, which can be classified in terms of length and complexity^6,7^. The shortest ones, the A-songs, consist of a single rattle. B-songs are initiated by a rattle and followed by a slower phrase, containing a more complex whistle syllable. Finally, C-songs contain more than two phrases in a sequence, consisting of both rattles and whistles (see Fig. 1 for illustration). Studies on limited sample sizes showed that A- and B- songs play an aggressive function in male-male interactions, while C-songs are important in sexual attraction, as they evoke stronger responses of females than of males^6,7^. The Aquatic Warbler features exceptional reproductive biology. Unlike any other *Acrocephalus* warbler it has a promiscuity breeding system without pair-bonds and with female-only parental care, and lacks plumage sexual dichromatism^8^. Intense sperm competition is inferred^9,10^. Therefore, the role and evolution of Aquatic Warbler song appears to be of special interest. In particular, as for some other *Acrocephalus* warblers, in the Aquatic Warbler song might be a crucial factor affecting mate choice by females^11^. Additionally, it is expected to be a signal of only genetic male quality, as opposed to e.g. quality of chick provisioning in biparental care species.

### Aims of the study

In the present study, we tried to fill the most important gaps in the knowledge about the Aquatic Warbler song. First, we analysed long recordings of individually marked males to find out how different song characteristics are related to each other. Second, we estimated, for the first time, the song phrase repertoire of the Aquatic Warbler with a new tool, the Koe software, which allows for computer-aided classification of syllables when repertoires are large. We tested whether the estimated phrase repertoire size is predicted by the number of different whistles and (or) different rattles, as these two song components are included in the A-, B- and C-songs in varying proportions, and, therefore, may have different functions^6,7^. Furthermore, we estimated the real phrase repertoire size of males with methodology derived from species diversity ecology, where basic song units are treated as individuals caught during species biodiversity sampling^10^. Finally, we tested whether and how the phrase repertoire size is linked with features characterising the way of singing: song rate, phrase duration, switching phrase types, and which of the three song types (A, B or C) best explains its variation.

## Results

### General characteristics of the sound material and song output

In total, recordings of 40 focal males were analysed. We aimed at recording 10 min per male, but this was not always possible. We collected at least 10 min of recording for 29 males. On average ($\overline{x }$ ± SD) we obtained 703 ± 278.1 s of recording per male, with an average of 235 ± 113.7 phrases per male. The analysed recordings were collected from a single bout.

**Figure 2:**
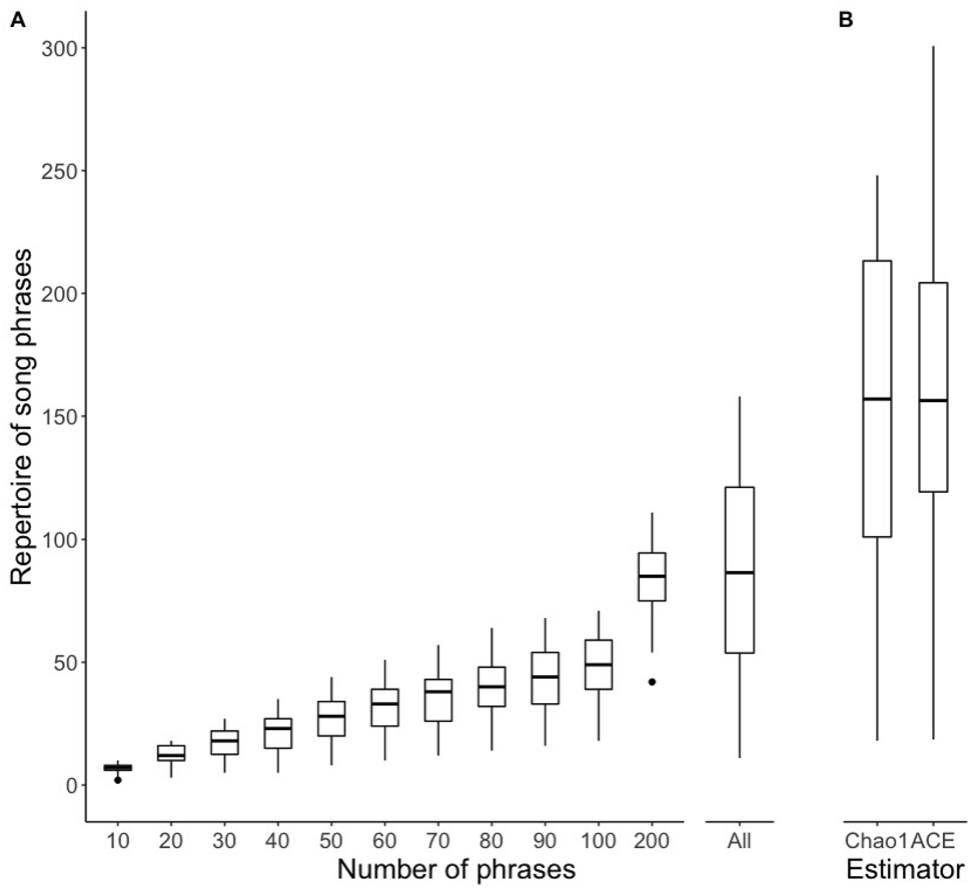
Song phrase repertoires of the Aquatic Warbler males. (**a**) Enumerated phrase repertoire as a function of the number of phrases from the start of a recording. Because the number of song phrases recorded from each male varied, the sample size for the enumerated phrase repertoire was N = 40 for 10–20 phrases, N = 39 for 30–40 phrases, N = 37 for 50–100 phrases, and N = 23 for 200 phrases. The enumerated phrase repertoire based on all phrases recorded (range 25–648) for each bird was calculated for all the 40 males. (**b**) Average phrase repertoire size estimated with the Chao1 and ACE estimators, based on all the 40 males.

We found between 11 and 158 different phrase types per male ($\overline{x }$ ± SD: 88 ± 40.5), but the lowest values came from the least-recorded males. When we considered only the males recorded for ≥ 10 min, the enumerated average phrase repertoire size increased to 99 ± 37.7 per male but still showed a broad range between 16 and 158 different phrases (Fig. 2). All the recorded males produced rattle and whistle phrases, while the mixed rattle-whistle category was not registered for 13 males (33%). All three song types, A, B, and C, were recorded from all except two males. In recordings of two males, we did not find B-songs, but for these males we had the shortest recordings of 49.5 s and 58.1 s, and recorded only 25 and 41 song phrases, respectively.

The general characteristics of the recordings and songs are presented in Table 1.

**Table 1:** Basic parameters of the Aquatic Warbler *Acrocephalus paludicola* recordings (N = 40 birds).

Song variable	Range	$\overline{x }$ ± SD
Duration of recordings per male (s)	49.6–1450.3	703.6 ± 281.65
Number of song phrases per male	25–648	235.4 ± 115.14
Number of whistle phrases per male	6–290	87.8 ± 52.65
Number of rattle phrases per male	19–340	142.2 ± 64.24
Number of rattle-whistle phrases per male	0–24	5.1 ± 6.61
Number of A-songs	1–118	44.4 ± 28.44
Number of B-songs	0–388	39.9 ± 61.09
Number of C-songs	3–50	22.7 ± 12.34
Song duty cycle (%)*	3.3–43.9	16.9 ± 7.21
Ratio of whistle to rattle number	0.21–1.13	0.61 ± 0.189
Ratio of whistle to rattle duration	0.31–1.19	0.73 ± 0.245
Mean whistle phrase duration (s)	0.35–0.86	0.52 ± 0.119
Mean rattle phrase duration (s)	0.29–0.73	0.44 ± 0.089
Mean rattle-whistle duration (s)	0.32–2.52	0.89 ± 0.349
Enumerated repertoire size of phrase types	11–158	87.5 ± 40.54
Phrase type switch rate**	0.52–1	0.89 ± 0.087
Category phrase switch rate**	0.33–0.86	0.59 ± 0.111

*Percentage of time spent singing in the total recording time.

**Switch rate is the number of switches from one phrase (category) type to a different one, divided by the total number of possible switches within a recording, which is equal to the number of phrases minus one. The switch rate may range from 0 to 1, where 1 means that a male switches to a different phrase or category type every phrase.

We found a significant correlation between the duration of a recording and the number of phrases recorded (*r*_s_ = 0.65, *p* < 0.001). The enumerated phrase repertoire size correlated more strongly with the number of phrases recorded (*r*_s_ = 0.83, *p* < 0.0001) than with the duration of a recording (*r*_s_ = 0.57, *p* = 0.001). Interestingly, the enumerated song phrase repertoire was not significantly correlated with the song duty cycle, understood as the percentage of time spent singing (*r*_s_ = 0.05, *p* = 1.0), while it correlated positively with the total time spent singing (*r*_s_ = 0.72, *p* < 0.0001). The enumerated repertoires of whistles ($\overline{x }$ ± SD, 33.2 ± 16.56) were on average smaller (paired t-test, *t* = -4.10, df = 78, *p* < 0.0001) than those of rattles (53.1 ± 25.76), and were significantly correlated (*r* = 0.79, *p* < 0.0001).

The Aquatic Warbler males sang quite variably and on average switched between different phrase categories slightly more often than every second phrase, and between phrase types slightly less often than every second phrase (Table 1). However, we found that the enumerated phrase repertoire size did not significantly correlate either with the category switch rate (*r*_s_ = 0.07, *p* = 0.654) or with the phrase type switch rate (*r*_s_ = 0.18, *p* = 0.243). Therefore, the song style described by these two variables (Table 1) did not directly reflect the phrase repertoire size.

**Figure 3:**
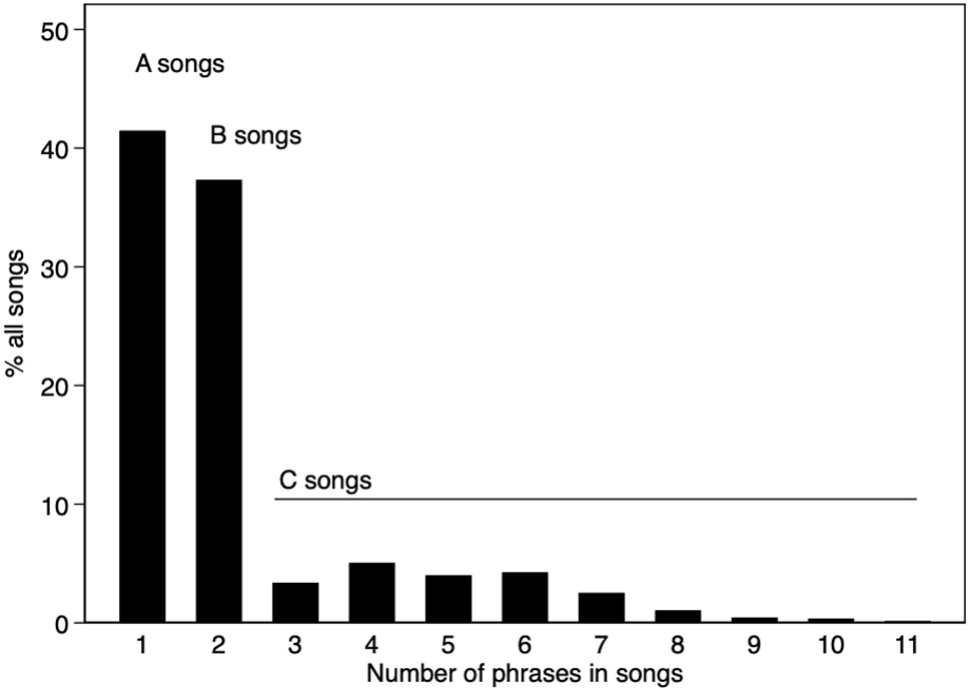
Percentage of A-, B- and C-songs recorded for all the 40 males, shown by the number of phrases per song.

The song phrases recorded for the 40 males were produced as single phrase A-songs (42%), double-phrase B-songs (37%) or three-and-more-phrase C-songs (21%). The C-songs contained from 3 to 11 phrases (Fig. 3).

These statistics indicate that the phrase repertoire found in the recordings depends not only on the duration of a recording but also on how many phrases are sung and on the style of singing (i.e., relative numbers of A, B and C songs). The enumerated phrase repertoire size was positively and significantly (Bonferroni-adjusted significance level) correlated with the number of C-songs recorded (*r*_s_ = 0.85, *p* < 0.0001), but not with the number of A-songs (*r*_s_ = 0.21, *p* = 1) or the number of B-songs recorded (*r*_s_ = 0.35, *p* = 0.146). The rattle repertoire was not significantly correlated with the number of A-songs (*r*_s_ = 0.31, *p* = 0.306) and B songs (*r*_s_ = 0.39, *p* = 0.074), while it was significantly correlated with the number of C-songs (*r*_s_ = 0.76, *p* < 0.0001). Likewise, whistle repertoires were correlated significantly only with the number of C-songs (*r*_s_ = 0.88, *p* < 0.0001), but not with B- (*r*_s_ = 0.25, *p* = 0.704) or A-songs (*r*_s_ = 0.06, *p* = 1).

To summarise, we found that the overall phrase repertoire size was explained more by rattle than by whistle phrase type variation, and that only the number of C-songs correlated with the enumerated phrase repertoire (also when repertoires of whistles and rattles were treated separately).

### Enumerated vs estimated real phrase repertoire size

**Figure 4:**
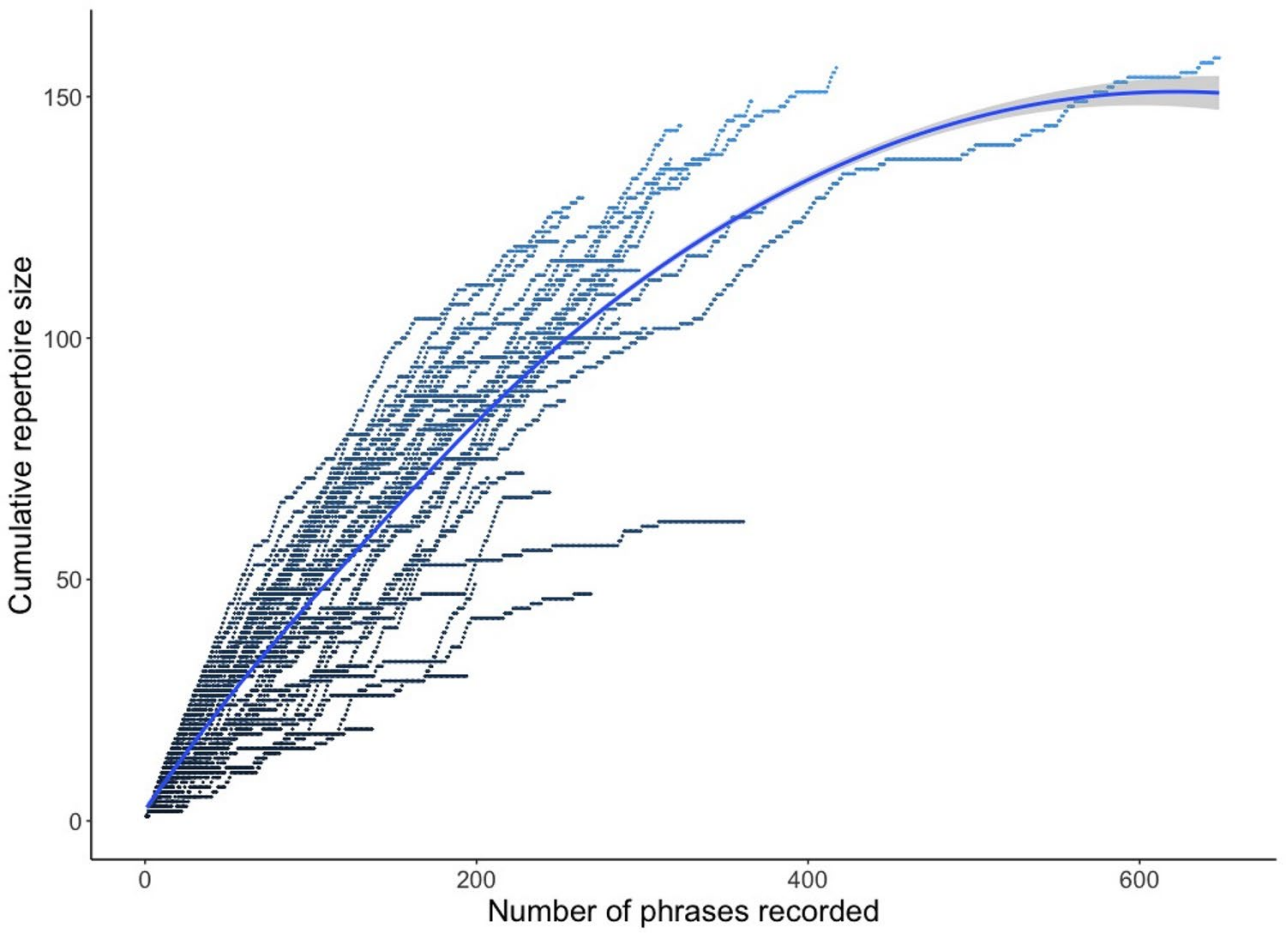
The cumulative enumerated phrase repertoire sizes for all the 40 males. The fitted line presents the average expected values (loess function, span = 0.8).

**Figure 5:**
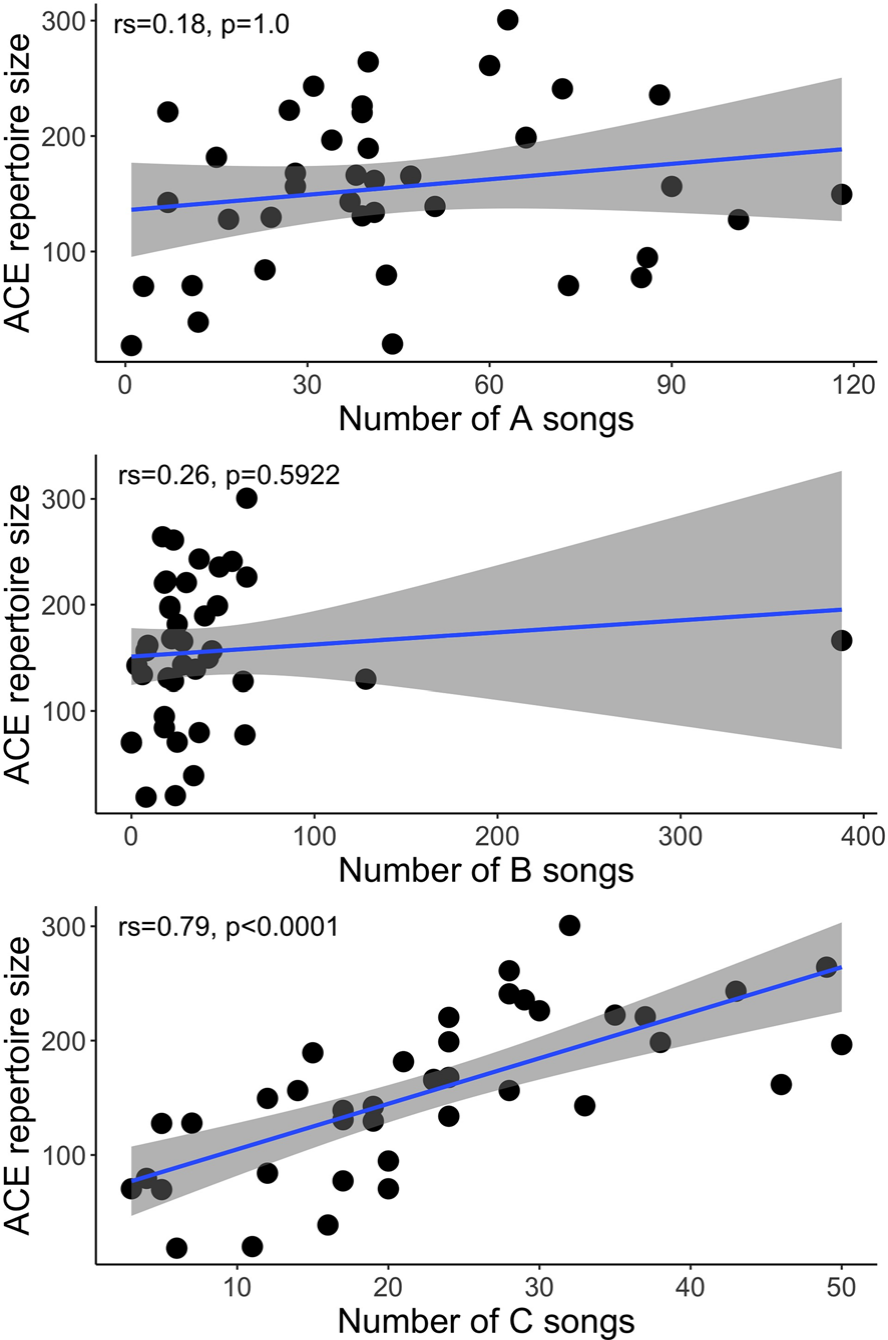
Correlations between numbers of A-, B- and C-songs recorded from males and the ACE, one of the best estimators of the entire phrase repertoire size.

The basic data on the enumerated phrase repertoire size presented above and the relationship between the enumerated phrase repertoire size and recording parameters as well as the style of singing strongly suggested that the real phrase repertoire of a male could be much larger than the phrase repertoire counted within recordings. The enumerated phrase type repertoire size based on small subsets of consecutive songs yielded underestimated values (Fig. 2). For example, when we compared the first 10 phrases and the first 100 phrases of the same males for which we had at least 100 phrases recorded, we obtained the average repertoire sizes ($\overline{x }$ ± SD) of 6.9 ± 2.00 and 46.6 ± 13.93 phrase types, respectively (paired t-test: t = − 18.32, N = 37, *p* < 0.0001). This discrepancy was still present when we compared the first 100 and 200 phrases for the males with ≥ 200 phrases recorded: 47.5 ± 14.67 vs. 82.6 ± 17.77 phrase types (paired t-test: t = − 17.30, N = 23, *p* < 0.0001). More details on how the sample size relates to the enumerated phrase repertoire size are presented in Fig. 2. As shown in the cumulative plot (Fig. 4), the rate of increase in the number of different phrase types in the repertoire varied between males. Estimation of the phrase repertoire sizes with a species richness paradigm yielded ($\overline{x }$ ± SD) 150.1 ± 66.7 for the Chao1 estimator and 155.5 ± 69.5 for ACE, with ranges of 18.1–248.1 and 18.5–300.7 phrases, respectively. Both of these repertoire estimators suggested that even with recordings of 10 or more minutes per male we still did not recorded the full repertoires (Fig. 2). The correlation between the number of A-, B- and C-songs and the estimated phrase repertoire yielded only the number of C-songs as a significant predictor (Fig. 5, Table 2).

**Table 2:** A linear regression model explaining the estimated repertoire size of Aquatic Warbler males (the ACE estimator) with the number of A-, B- and C-songs sung by the males (N = 40).

	Estimate	SE	t	*p*
(Intercept)	42.57	20.869	2.04	0.049
A-songs	0.43	0.277	1.56	0.128
B-songs	0.09	0.129	0.67	0.497
C-songs	3.98	0.637	6.24	**< 0.0001**

Significant values are in [bold].

To summarise, the true phrase repertoires of Aquatic Warbler males are likely larger than the enumerated ones, even when the latter are based on a long recording of around 10 min. The phrase repertoire size is formed by variation in both whistles and rattles, but the repertoire of rattles appears to be larger than that of whistles. Finally, only singing C-songs is related to the presentation of the real phrase repertoire size (Fig. 5). Therefore, Aquatic Warbler males can convey information about their repertoire complexity in a relatively short sample of the song.

## Discussion

### What is the repertoire size of the Aquatic Warbler?

Simple counts of song phrases detected with the help of Koe^12^ revealed that the Aquatic Warbler males had relatively large enumerated repertoires, reaching on average 99 different phrases when recorded for ≥ 10 min. However, plotting cumulative curves of distinct phrases indicated that even our longest recordings did not reveal the entire phrase repertoires of all the males. The estimation of the phrase repertoire sizes with a species richness paradigm showed an average value around 150–155 (depending on the model used for estimation). The phrase repertoire sizes also showed high between-individual variation with an estimated range between 18 and 300 different phrases. It was the diversity of rattles, rather than the diversity of whistles, that contributed more to phrase repertoire variation, despite their being positively correlated. Since we did not have very long recordings, we cannot be completely certain how well the estimators worked. Nevertheless, even based on the enumerated repertoire sizes, it is clear that the repertoires are large and that males vary in song complexity to a great extent. Our results also indicate that the estimated repertoire sizes were larger the enumerated ones, although we should make a reservation that our samples (i.e. numbers of phrases recorded) might have been too small to capture the complete repertoires^13^. These findings shed new light on the singing abilities of the species. Although male Aquatic Warblers are not continuous singers, their song variability is impressive, and the phrase repertoire is larger by an order of magnitude than the previously suggested values^14^. The most surprising result for us was, however, not the size of the phrase repertoires, but the fact that both whistles and rattles were highly variable. The earliest descriptions of Aquatic Warbler song did not explore this aspect at length. However, it appears that in the past, it was whistles that were treated as diverse song units responsible for building repertoires, while rattles were considered to be largely invariable (Andrzej Dyrcz, personal comm.). Our results show that this is certainly not the case. We suspect that one of the reasons that variation in whistles was over-emphasized in earlier work is that differences in whistles are easier to discern than differences in rattles, both in terms of discriminating sounds by ear as well as visually on sonograms. Rattles consist of very short units produced at a distinctly faster rate than whistles. Moreover, they often differ from each other with respect to hard to detect, shifted peaks in maximum amplitude in the frequency domain. Some of rattles differ also in the temporal pattern, which is associated with the rhythm of pulses (see Fig. 1). Undoubtedly, differences in hearing abilities between birds and humans are a factor that makes interpretation of the results difficult, and future studies should test how Aquatic Warblers respond to signal variation that we described here^15,16^. As A-songs consist of single rattles and they were suggested to deter male rivals, it was probably easier to consider that they were not as diversified as whistles, which mostly appear in C-songs directed at females^6^. The possibility of measuring a large set of characteristics and using it for classification with a tool such as Koe^12^ enabled us to catch this hidden variation. We think that this is a good example showing that new bioacoustics tools allow for new findings that are more representative and objective.

To sum up, song-phrase repertoires of Aquatic Warbler males are much larger than previously thought, and both whistles and rattles contribute to the repertoire size, with a significantly larger number of different rattles than that of whistles.

### How does the organisation of song output relate to the song phrase repertoire size?

Another question that we wanted to address in this study was whether and how the phrase repertoire size is related to the organisation of song output. In other words, do the males with larger (and likely more attractive) repertoires sing differently, or is repertoire variation merely limited to the number of different song units within the vocal output? The style of song output is often variable and in theory this variability could be linked to male quality. In fact, it is explained by two characteristics of the song output: syntax and rate. Even with a small repertoire, a singer may sing in a diverse way, switching between different units as often as possible, e.g. AB AB AB AB AB… This singing pattern is not equal to producing AB CD EF GH IJ…, where each song unit produced is different, although the switching pattern between the different units is exactly the same. In addition, both sequences could be—for example—produced during 10, 100 or 1000 s, i.e. sung with a different rate. In some acrocephalids, e.g. in the continuously singing Marsh Warbler (*Acrocephalus palustris*) there is a correlation between faster song rate and presentation of a larger repertoire^17^. However, this was not the case in the Aquatic Warblers. Males with larger repertoires did not sing faster and did not switch faster between different phrase categories or types. We found that it is the higher level of song organisation, i.e. producing A-, B- and C-songs that is important. Correlations between the number of these songs and both the enumerated and the estimated phrase repertoire size unequivocally revealed that only C-songs play a crucial role in presenting the repertoire size. This result adds support to earlier findings suggesting that C-songs are important in mate attraction^6,7^.

Here, we complement this knowledge by showing that C-songs are highly diverse in both content and duration, and that this leads to clear differences between individuals (Fig. 4). Such variation in repertoire size was shown to affect female choice in other acrocephalids. For example, in the Sedge Warbler (*Acrocephalus schoenobaenus*) it was demonstrated that the song repertoire size has a significant effect on the pairing date^18^. In the polygynous Great Reed Warbler (*Acrocephalus arundinaceus*), males with larger repertoires attracted more females for copulation and sired more offspring than social mates with smaller repertoires^2^. This latter study also demonstrated that females engaging in extra-pair fertilisations benefit from having genetically better offspring. Therefore, the repertoire size and its individual variability in the Aquatic Warbler indicate that in this species song phrase repertoire could have evolved as an elaborated sexually selected trait, especially in the light of the promiscuous mating system.

Our analyses with the repertoire size estimators also show that reliable evaluation of the repertoire size depends more on the number of recorded song units than on the duration of recording. If a male sings continuously, a relatively short singing period could be used for evaluating the repertoire size. In other words, a few long and complicated C-songs are good predictors of a large repertoire. A similar result was previously found for the Marsh Warbler, in which two-minute-long recordings were used to estimate the repertoire size with capture-recapture and species-richness models, and the repertoire size correlated with male nesting success^17^. In both species males apparently differ in the repertoire size, which can be an honest signal of male quality^2^. The song communication system of the Aquatic Warbler should therefore be considered as very flexible and efficient. Within a short time, males are able to produce many simple A- and B-songs, with a high rate of production being more effective at deterring other males, and to smoothly switch to longer and complicated C songs, which are addressed to females searching for high-quality partners.

Finally, we want to point out one more observation. The comparison of thousands of phrases indicated that the Aquatic Warbler males were more flexible in producing different rattle than different whistle phrase types. As mentioned earlier, different rattle phrases often seemed to be created by slight modification of parameters such as the note rate or dominant frequency, which changed in a continuous rather than discrete way; moreover, these parameters often changed within a phrase (see examples: Fig. 1j,k,m–o). This unexpected large variation of rattle phrases, especially presented in C-songs, could suggest that males singing these longer songs toward females use an alternative mechanism of generating song variation. However, it is worth remembering that we do not know how Aquatic Warblers perceive the variability described here and that some birds might perceive graded variation in a categorical manner^19^. If we consider that the classic repertoire relies on physical entities remembered in the bird’s brain and presented (sung) in a particular context, the alternative mechanism could be flexible modification of such units to increase variation of the final output. It is a hypothesis, but the comparison of hundreds of whistles and rattles clearly showed that different whistles usually had different frequency track and duration, while different rattles quite often resembled more graded variation on the same theme. These variations do not appear when a male sings several A-songs in a sequence, but they are present if longer C-songs are sung.

## Concluding remarks

As in the Aquatic Warbler both sexes are promiscuous, there is no paternal offspring care and no sexual plumage dichromatism, the repertoire size is expected to be an important sexually-selected trait and hence the species appears a good model to study sexual selection in songbirds. Our results will therefore inform future studies relating song features to female mate choice and male quality- as well as fitness-related traits in this species and in other songbirds with a polygynous or promiscuous mating system. In further studies, it will be undoubtedly crucial to obtain long recordings and multiple recordings of the same individually marked males. With such data, it will be possible to verify how large the Aquatic Warbler repertoires could be and whether males present song complexity consistently or sing differently under different circumstances.

## Material and methods

### Study area and sampling

The study was conducted in 2017–2018, in one of the core breeding sites of the Aquatic Warbler—the Biebrza Valley, Poland. This area holds about 25% (c. 2700 singing males) of the global population. Three study areas were used (central positions: Ławki, N 53° 17′ 11.4″, E 22° 33′ 49.2″; Szorce N 53° 17′ 34.8″ E 22° 37′ 15.9″; Mścichy N 53° 25′ 41.7″ E 22° 30′ 17.0″), with two 10–20-ha study plots in each study area.

Males were detected by ear and caught with mist-nets. Each bird was marked with unique metal and colour rings. All the males were reproductively active i.e., in their second calendar year or older, as identified by plumage. The males were also blood-sampled for the purposes of another study. The recordings were made between 1 day and about 1 year after blood-sampling.

### Ethics declarations

All procedures were conducted in accordance with Polish and EU law and did not require approval by the Local Ethical Commission because no experimental procedures were performed in the study (according to the Polish Act on Experiments on Animals). The fieldwork procedures were conducted under permits from the Regional Directorate for Environmental Protection in Białystok (no. WPN.6401.244.2016.MN) and the Polish Ministry of Environment (no. DOP-WPN.286.38.2016.AN). This study has been designed and performed in accordance with the ARRIVE guidelines^20^.

### Recording

Songs of the individually marked Aquatic Warbler males were recorded between 16:15 and 21:20 h, from 28 May to 29 July, 2018. The geographic position of each bird was stored using a GPS receiver. The code of the colour rings was identified with an 80-mm-lens and 20–60 magnification scope. The songs were recorded with a Marantz PMD 661 digital recorder and a Telinga Pro 7 microphone mounted in a Telinga Universal parabola, as 48 kHz/16 bit PCM .wav files.

In total, 45 males were song-recorded. Aquatic Warbler males have a narrow window (around dusk) during which songs can be recorded effectively. Combined with remote and difficult habitat, the time necessary for identifying a male with a scope and the large home ranges of males, this makes data collection slow. We suppose that females might "sample" males in a similar manner, in the sense that the cost of sampling is time. If it is possible to get reliable information about the repertoire size in a shorter time, there is no point in spending more time on it.

### Sound analysis

The .wav files were down-sampled to 22.05 kHz/16 bit and displayed on screen with the Hann window type, 1024 FFT sampling and 50% overlap, which resulted in 23.2 ms × 21.5 Hz spectrogram resolution. In discontinuous singers, the repertoire size is determined as the number of different strophe types. However, strophes of the Aquatic Warbler song are built of series of notes, called phrases, which are basic units of song production. Therefore, by manual segmentation in Raven Pro 1.6 (Cornell Lab of Ornithology), songs of each individual were categorised into phrases^6,7^ and the categorisation was then saved in a comma-separated (.csv) file. We also recorded the start and end time of a phrase in relation to the beginning of recording, as well as the minimal and maximal frequency. Each phrase was assigned to one of the three categories: whistle, rattle or rattle-whistle, based on visual and auditory inspection (Fig. 1). Two persons did the categorisation, one experienced (TSO) and one naïve observer (who works neither on birds nor on song). Both had 100% agreement on assignment to the three categories mentioned. In further analysis, we excluded five males with fewer than 10 phrases recorded. Whistle phrases were built of tonal sounds, which were usually repeated a few times in a series. Typically, whistle phrases were uniform, i.e. all the syllables within a phrase were basically the same (Fig. 1a,b), and phrases were rarely built of a single whistle (Fig. 1c–e), a few whistles similar in frequency and duration but slightly different in detail (Fig. 1f,g), or a series of syllables which continuously changed within a phrase (e.g. decreased in frequency; Fig. 1h,i). Rattle phrases consisted of short, noisy syllables with a wide frequency band (Fig. 1j–o). Rattles were formally much more structurally variable than whistles. Syllables within rattles sometimes contained a tonal component (Fig. 1j) but sometimes they did not (Fig. 1k); they were uniform (Fig. 1l) or changed (Fig. 1m–o) in the overall and/or dominant frequency. Pauses between syllables forming rattle phrases were found to be uniform (Fig. 1j,l), or to decrease or increase with time (Fig. 1k,n). Such features change rattle rate perception to the human ear and were characteristic only to this category of phrases. In rare cases (2.1% of all phrases recorded), we also noticed complex, longer phrases (rattle-whistles), which always started with a rattle and smoothly turned into a whistle, and were always sung together in the same way (Fig. 1p,q). It is worth noting that Fig. 1 shows single examples of different phrases and does not reflect how often they were sung. In that figure, the panel ‘Song categories’ presents three categories of syntax (how phrases are linked), while the two remaining panels show examples of phrases which were classified as different types that built the repertoires. Some of these examples are more representative in terms of structure, but this did not affect the analyses shown here.

The main difference between the variability of whistles and rattles was that whistles of a certain type differed distinctly from other types in the tonal course of sound, which was easy to distinguish visually on sonograms (i.e. it was possible to identify the same whistle types in repertoires of different individuals; Fig. 1a–i, Fig. S2). In the case of rattles, new sonically distinguishable units seemed to arise by changing one or more of the continuously variable parameters, such as the syllable rate, dominant frequency of consecutive notes, number of notes forming groups within a rattle, etc. (see Fig. 1j–q and examples in Fig. S2). To sum up, rattles were found to be much more variable than whistles.

### Classification of phrases into types

Classification of all the three phrase categories into discrete types was done with the Koe software^12^. Recordings of each male were uploaded into the Koe server and then the corresponding text files (.csv) with phrase selections from Raven Pro were imported into Koe. Each of the imported phrase selection was assigned a unique ID and the phrase category (whistle, rattle, rattle-whistle) was included as the ‘Family’ column (see the Koe manual).

In the next step, we extracted unit (i.e. phrase) features in order to obtain data matrices for further comparison. Based on the visual inspection of phrases during manual selection, we decided to extract only such features which enabled assigning phrases automatically to the same category if they sounded consistently the same. Therefore, we chose all the features related to overall frequency and within-phrase changes of frequency and/or amplitude. We omitted all the features that may reflect the quality of recording (i.e. related to energy or power measurements), as all the recordings were obtained from a short distance and were of comparable and very good quality. We also omitted overall phrase duration features, as phrases often contained different numbers of syllables and differed by duration; while still consisting of the same shape syllables and sounding similarly, they were just shorter or longer. The final choice of features extracted was made after several tests in which we initially used all features as well as several combinations of different features. The full list of Koe features extracted and their descriptive statistics used for building the data matrices is presented in Table S1.

We used the prepared data matrices to construct ordination with the t-SNE (t-distributed Stochastic Neighbour Embedding) and the PCA (principal component analysis) pre-processing Koe method. The combination of these two methods allows for retaining the overall variance of the dataset (characteristics of song phrases) with nonlinear dimensionality reduction allowing for efficient 2D data visualisation. Before we selected specific parameters for the final analysis, we ran several test by varying the available parameters to find out which set of parameters provided the best results, i.e. clustered together those phrases which sounded and looked similar on spectrograms. Finally, for all the recordings we constructed two-dimensional ordination with t-SNE perplexity parameter of 40 and the number of iterations equal to 10,000. In the next step, in Koe we calculated similarity of phrases for each male separately. The similarity is a value from 0 to *N*, where *N* is the total number of units in an analysed database of sound units. The Koe similarity works as a rank scale. For example, if one compares 100 units (the numbers go automatically from 0 to 99), the unit with 0 similarity is the least similar to the unit with 99 similarity, while 5 and 6, or 83 and 84 are the most similar to each other, as they are neighbours on the scale. On the other hand, as with all rank scales, there is no information about similarity or dissimilarity distance between such values, hence 5 could be much more similar to 6 than 6–7, or when we compare similar 100 phrases, they will still be assigned to 0–99 similarity categories. However, this tool enables quick comparison of many units, as seeing and listening to neighbouring sounds in the unit table is easy. While assigning each phrase to the category we first visually checked the two-dimensional ordination, and with the use of the available tools (lasso, zoom, autoscale) we added different labels to each phrase or group of phrases which separated as different on the ordination map (Fig. S1). After the whole material has been analysed by one naïve observer, the second observer (TSO) checked all the ordination maps again with the use of unit table and Koe exemplar views (Fig. S2).

To summarise, each phrase within a male repertoire was classified to one of the three categories (whistle, rattle or rattle-whistle) and to a discrete types, which received unique number. Phrases of the same type were placed closer on the ordination map based on 23 feature measurements (Table S1), t_SNE ordination and judgement of two independent observers, and formed visual aid in Koe.

### Characterising song output

Recordings of each male were characterised by duration(s) and the total number of phrases that were assigned to the three general categories (whistle, rattle, rattle–whistle) and to several unique types (forming the phrase repertoire) within these categories (Table 1). Because the mixed rattle-whistle phrase category was relatively rare, not found in all the males and acoustically resemble a whistle, it was treated together with the whistle category in some singing style characterisations described below. If we treated rattle–whistles as B-songs consisting of a rattle and a whistle (with an arbitrary choice of the place where they are separated), this did not affect results, including the repertoire size and song presentation analyses. Based on the classical approach of Catchpole and Leisler^6,7^, we also characterised the song output of each male by showing how he uses the song phrase to compose songs into three syntactical categories: simple A-song (rattle), slightly more complicated B-songs (rattle + whistle) and the most complicated and longest C-songs.

Finally, we characterised the singing effort and style of the males (Table 1). We calculated the duration of phrases (total as well as by categories and types), which could be treated as a song duty cycle measure (when calculated as a fraction in the whole duration of recording). A male may sing the same phrase category in a sequence (whistle–whistle–whistle…) or immediately switch between categories (whistle–rattle–whistle…). Similarly, the type of phrase could be repeated several times in a row (eventual variety) or switched with every new phrase in a sequence (immediate variety). To characterise these two song patterns, we calculated phrase category switch rate and phrase type switch rate, dividing the number of switches by the number of all the possible switches (equal to the number of phrases in the sequence minus one). Hence, both the category switch ratio and phrase type switch ratio range from 0 to 1, where 1 indicates that each following category or phrase type is different than the preceding one in a song sequence.

### Estimating the real repertoire size

We found that Aquatic Warbler males produced many different types of phrases with variation suggesting that the repertoire size could be very large. In long recordings with more than 100 or even 200 phrases recorded we still were finding new phrase types after initial 100 or 200 phrases. This was expected, as bird species with song organisation close to the continuous song often have large repertoires and deliver syllables with heterogeneous probability^10,17^. Preliminary analyses of correlations between the enumerated phrase repertoire size for the whole recording (full sample) and the phrase repertoire size based on subsamples (from 10 to 200 phrases) indicated that the enumerated phrase repertoire size does not reflect the real phrase repertoire size that a male may reveal during longer singing. Therefore, to estimate the phrase repertoire size, we used measures derived from species diversity ecology^21^. The most promising methods in the context of repertoire size estimation are those which treat syllables or other repertoire units (phrases in our case) as ‘species’ and use abundance or incidence-frequency data for measuring such ‘species’ richness. We used the SPADE (Species Prediction and Diversity Estimation) software^22^. We prepared data according to the SPADE manual by sorting the number of occurrences of each phrase type in descending order. We choose the default ‘Cut off point = 10’ for ACE and ICE estimators and set the number of bootstraps to 1000. The SPADE automatically calculates nine different estimators/models and outputs an estimate, its standard error and 95% confidence intervals. Among SPADE estimators are equivalents to those used by Garamszegi et al.^10^ for testing estimation of song complexity on 18 bird species, including M(t), M(h) and M(th) models, which were shown to be best for species with large repertoires^23^.

We thoroughly inspected the enumerated repertoire sizes and estimated repertoire sizes calculated on different subsets of original data. Based on this and on theoretical assumptions of the different estimating methods, we finally chose the estimates which worked best in terms of giving the closest estimate based on the first 100 phrases of the enumerated repertoire for the whole male dataset with over 200 phrases recorded (N = 23). We found that the best estimators, which yielded very similar values, were Chao1 and ACE. However, all nine estimators gave highly correlated results (r varied between 0.84 and 0.99, all *p* < 0.001). Chao1 returns an estimate of species (here phrase) richness based on a vector or matrix of abundance data, while ACE is based on the concept of sample coverage, which was originally developed for cryptographic analyses by Alan Turing^23^. The Chao1 approach uses the number of singletons and doubletons (here, phrase types found in recording only once or two times) to estimate the number of undetected units. The ACE estimator separates phrases into abundant and rare groups and uses only the rare group to estimate the number of undetected species^22^. In our dataset, Chao1 had a slight tendency to overestimate the repertoire size, while ACE underestimated it. However, a comparison of the Chao1 estimates and ACE estimates with a paired t-test showed no significant difference (t = − 1.54, N = 40, *p* = 0.132). For illustrative purposes we use the ACE estimate throughout.

### Statistical analysis

We used parametric or non-parametric (in cases of non-normal distribution) tests to seek correlation between song characteristics (Spearman’s and Pearson’s correlation) or test for differences between compared groups (t-test or Mann–Whitney test). We used the Bonferroni correction to reduce the chances of obtaining false-positive results when multiple tests were performed. In order to evaluate the relationship of the singing characteristics with the phrase repertoire size we used linear regression models with the song phrase repertoire as the explained variable. We performed the analysis in the R environment v. 4.0.1 (R Core Team 2021).

## Supplementary Information

Supplementary Information.

## Data Availability

The datasets analysed during the current study are available from the corresponding author on reasonable request (Tomasz S. Osiejuk, email: osiejuk@amu.edu.pl).
