# Supplementary material for: Male song of the Aquatic Warbler, a promiscuous bird without paternal care, is more complex than previously thought
Source: Sci Rep. 2023 Apr 7;13:5714. doi: 10.1038/s41598-023-33001-9 (PMC10082015; doi:10.1038/s41598-023-33001-9)
Supplement: Supplementary file 1 — Supplementary Information. [file 41598_2023_33001_MOESM1_ESM.docx]

**Supplementary material**

**Figure S1.** Ordination map presenting classification of the Aquatic Warbler song phrases in the Koe software. Each of the colour symbols indicates a different whistle or rattle phrases from repertoire of male no. 6.

**Figure S2.** Exemplars window of Koe showing exemplary sonograms of the most frequent song phrases classified as different in the repertoire of male no. 6.

**Table S1.** Parameters of song feature extractions in Koe.


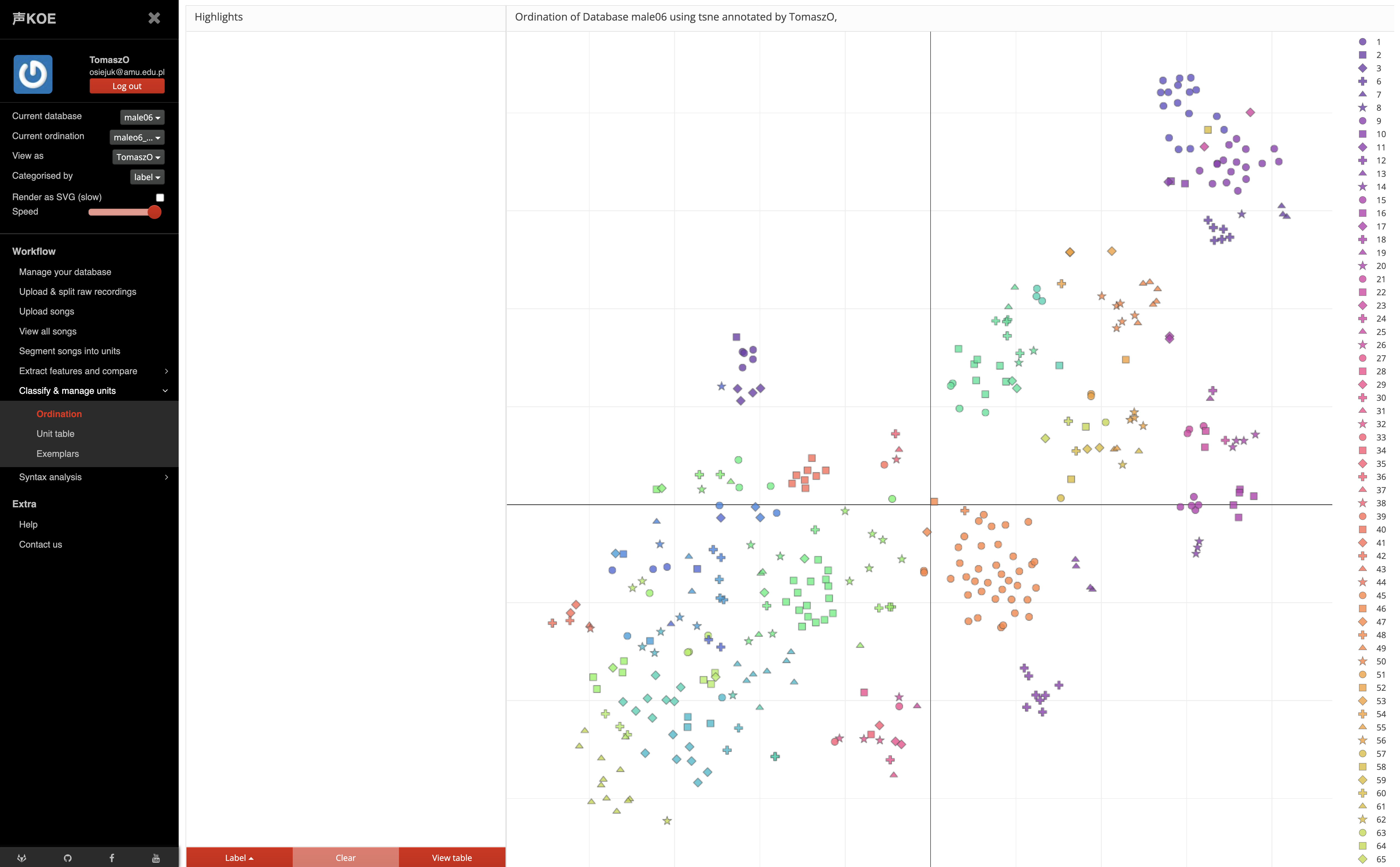


**Figure S1.** Ordination map presenting classification of the Aquatic Warbler song phrases in the Koe software. Each of the colour symbols indicates a different whistle or rattle phrases from repertoire of male no. 6.


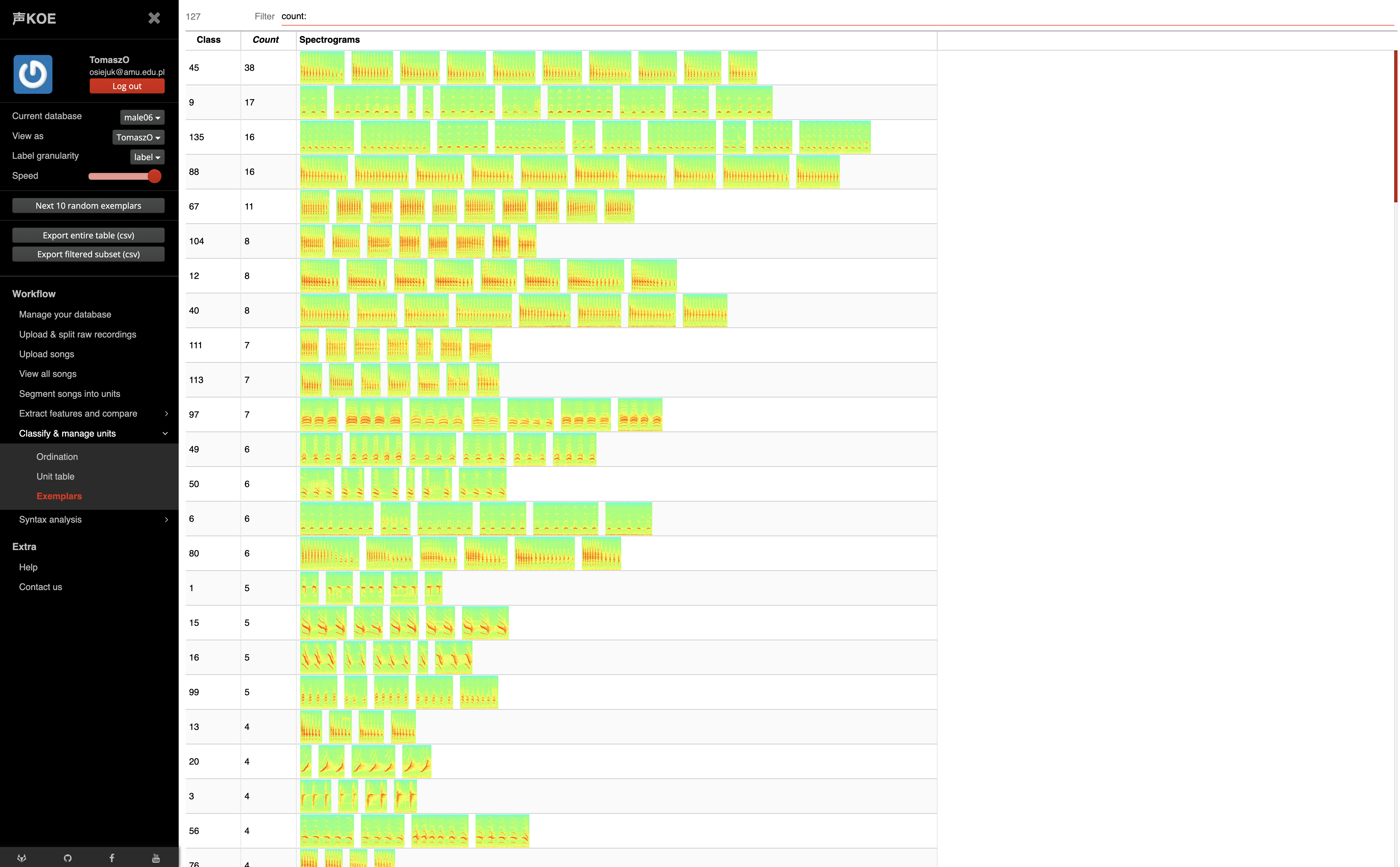


**Figure S2.** Exemplars window of Koe showing exemplary sonograms of the most frequent song phrases classified as different in the repertoire of male no. 6.

**Table S1.** Parameters of song feature extractions in Koe.

We extracted the following sound features for each phrase: spectral_flatness, spectral_bandwidth, spectral_centroid, spectral_contrast, spectral_rolloff, mfcc, max_frequency, frequency_modulation, amplitude_modulation, goodness_of_pitch, mean_frequency, spectral_continuity, dominant _frequency, spectral_flux, spectral_crest, spectral_skewness, spectral_kurtosis, spectral_decrease, harmonic_ratio, fundamental_frequency, mfc, mfc_delta, mfcc_delta2, log_attack_time.

For the aggregation of features we used the following KOE parameters: mean, median, std, min, max, variance, begin, end.
